# Legionella pneumophila Exploits PI(4)P to Anchor Secreted Effector Proteins to the Replicative Vacuole

**DOI:** 10.1371/journal.ppat.0020046

**Published:** 2006-05-19

**Authors:** Stefan S Weber, Curdin Ragaz, Katrin Reus, Yves Nyfeler, Hubert Hilbi

**Affiliations:** Institute of Microbiology, ETH Zürich, Zürich, Switzerland; Tufts University, United States of America

## Abstract

The causative agent of Legionnaires' disease, *Legionella pneumophila,* employs the intracellular multiplication (Icm)/defective organelle trafficking (Dot) type IV secretion system (T4SS) to upregulate phagocytosis and to establish a replicative vacuole in amoebae and macrophages. *Legionella*-containing vacuoles (LCVs) do not fuse with endosomes but recruit early secretory vesicles. Here we analyze the role of host cell phosphoinositide (PI) metabolism during uptake and intracellular replication of *L. pneumophila.* Genetic and pharmacological evidence suggests that class I phosphatidylinositol(3) kinases (PI3Ks) are dispensable for phagocytosis of wild-type L. pneumophila but inhibit intracellular replication of the bacteria and participate in the modulation of the LCV. Uptake and degradation of an *icmT* mutant strain lacking a functional Icm/Dot transporter was promoted by PI3Ks. We identified Icm/Dot–secreted proteins which specifically bind to phosphatidylinositol(4) phosphate (PI(4)P) in vitro and preferentially localize to LCVs in the absence of functional PI3Ks. PI(4)P was found to be present on LCVs using as a probe either an antibody against PI(4)P or the PH domain of the PI(4)P-binding protein FAPP1 (phosphatidylinositol(4) phosphate adaptor protein-1). Moreover, the presence of PI(4)P on LCVs required a functional Icm/Dot T4SS. Our results indicate that L. pneumophila modulates host cell PI metabolism and exploits the Golgi lipid second messenger PI(4)P to anchor secreted effector proteins to the LCV.

## Synopsis

The bacterium Legionella pneumophila causes Legionnaires' disease, a severe pneumonia. In the environment, L. pneumophila multiplies within amoebae. By inhaling contaminated water droplets, L. pneumophila is transmitted to the human lung and grows within immune cells (macrophages). Within amoebae and macrophages, L. pneumophila replicates with a similar mechanism by forming a membrane-bound compartment, the *Legionella*-containing vacuole (LCV). Formation of the LCV is not well defined, but requires that L. pneumophila injects proteins into the host cell via the intracellular multiplication (Icm)/defective organelle trafficking (Dot) secretion system.

Phosphoinositide (PI) lipids are central mediators of membrane dynamics in amoebae and mammalian cells. In this study, the researchers report that phosphatidylinositol(3) kinases (enzymes that add a phosphate residue to PIs) are dispensable for uptake of *L. pneumophila,* but affect the formation of the LCV, as well as uptake and degradation of mutant L. pneumophila lacking a functional Icm/Dot system. Icm/Dot–secreted proteins were identified which specifically bind to phosphatidylinositol(4) phosphate (PI(4)P), a marker of the Golgi organelle in the secretory pathway, which is also present on the LCV. These findings indicate that L. pneumophila exploits PI(4)P to anchor secreted effector proteins to the LCV, thus subverting host cell PI metabolism to establish its replicative niche.

## Introduction

In the environment, the gram-negative bacterium Legionella pneumophila colonizes biofilms and multiplies within various protozoa [1]. Upon transmission to the human lung, the bacteria replicate within alveolar macrophages, and may cause the severe pneumonia Legionnaires' disease [2,3]. To establish its replicative niche, L. pneumophila prevents the fusion of its phagosome with lysosomes [4], and recruits early secretory vesicles at endoplasmic reticulum (ER) exit sites [5]. The resulting *Legionella*-containing vacuole (LCV) is characterized by the ER marker calnexin, the v-SNARE Sec22b, and the small GTPases Arf1 and Rab1 [6,7]. Moreover, the LCV undergoes a transition from a “tight” to a “spacious” vacuole [8,9] and eventually matures into an acidic compartment [10], wherein the bacteria multiply independently of the bacterial intracellular multiplication (Icm)/defective organelle trafficking (Dot) type IV secretion system (T4SS) [11]. The Icm/Dot T4SS is a conjugation apparatus that is encoded by 25 different genes and is required for formation of the LCV [12,13], as well as for modulation of phagocytosis [14,15].

To date, more than 30 different Icm/Dot–secreted proteins have been identified as putative effectors, many of which form families of between two and six paralogs [16–23]. The precise function of most of these proteins is not known, owing at least in part to the fact that L. pneumophila strains lacking even multiple family members do not show a phenotype with regard to intracellular replication [18,20,22]. However, the inability of *icm*/*dot* mutants to direct phagocytosis and establish a LCV suggests that at least some Icm/Dot–secreted proteins interfere with host cell phagocytosis or vesicle trafficking. Indeed, the recently identified effectors LepA and LepB share homology with SNAREs and seem to promote the non-lytic release of vesicles containing L. pneumophila from amoebae [19]. The first Icm/Dot substrate to be functionally characterized, RalF, recruits the GTPase Arf1 to the LCV and acts as a guanine nucleotide exchange factor for the Arf family of small GTPases [16]. To subvert host cell trafficking, the large number of Icm/Dot–secreted proteins is likely organized in a complex spatial and temporal manner.

The metabolism of phosphoinositide (PI) lipids is pivotal for the regulation of membrane dynamics during phagocytosis, endocytosis, and exocytosis [24,25]. Depending on phosphorylation at positions 3, 4, and/or 5 of the D-*myo*-inositol ring, PIs recruit specific effectors to distinct membranes in a time- and organelle-dependent manner, thus coordinating intracellular membrane trafficking and actin remodeling, as well as receptor-mediated signal transduction. The central role of PI second messengers is exploited by a number of intracellular bacterial pathogens [26], e.g., Shigella flexneri [27] and Salmonella enterica [28,29] employ type III-secreted PI phosphatases to modulate host cell PI metabolism during bacterial entry and intracellular replication.

PI metabolism is well characterized in the social amoeba Dictyostelium discoideum [30,31], which supports Icm/Dot–dependent intracellular replication of L. pneumophila [32–34]. Here, we use a *Dictyostelium* strain lacking the class I phosphatidylinositol(3) kinase (PI3K)–1 and −2 (Δ*PI3K1/2*; [35]) to demonstrate a role for PI metabolism in phagocytosis, trafficking, and intracellular replication of *L. pneumophila.* Furthermore, we identify Icm/Dot–secreted proteins, which specifically bind to phosphatidylinositol(4) phosphate (PI(4)P), thus providing a mechanistic link between PI metabolism and the subversion of host cell trafficking by *L. pneumophila.*

## Results

### PI3Ks Are Dispensable for Phagocytosis of Wild-Type L. pneumophila

**Figure 1:**
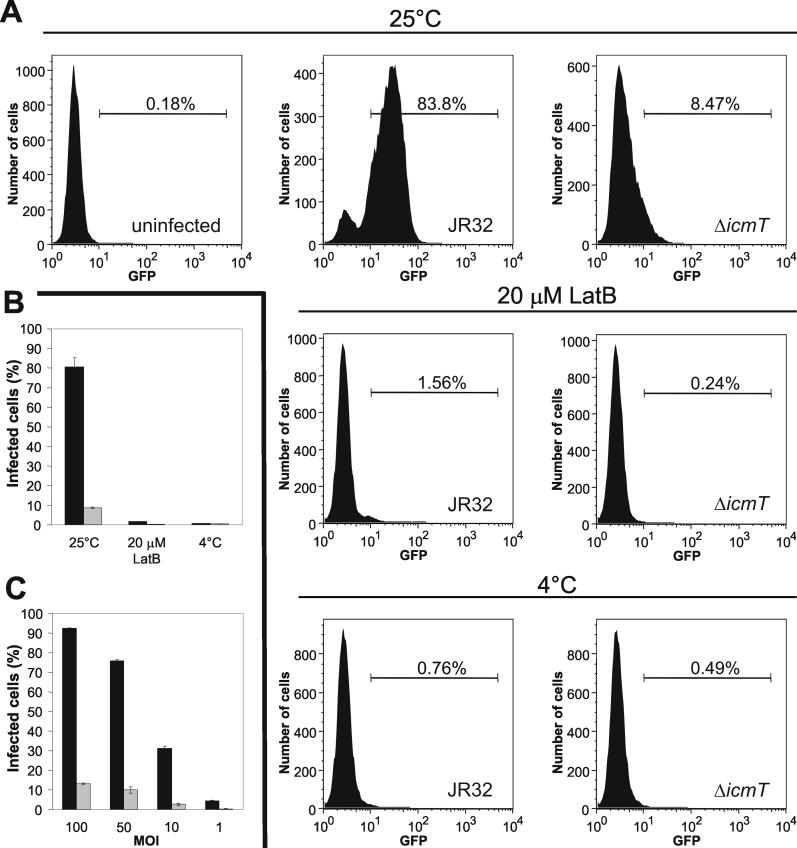
Phagocytosis of Wild-Type L. pneumophila or Δ*icmT* by *Dictyostelium* Phagocytosis of *gfp*-expressing wild-type L. pneumophila (black bars) or Δ*icmT* (grey bars) by *Dictyostelium* infected at (A and B) an MOI of 100 or (C) at the MOI indicated was analyzed by flow cytometry. The increase in GFP fluorescence (FL1, *x*-axis) indicates that, at MOIs ranging from 1–100, the number of wild-type L. pneumophila phagocytosed is about one order of magnitude higher than the number of Δ*icmT.* Phagocytosis is blocked by latrunculin B or incubation at 4 °C. (B and C) The data shown are the means and standard deviations of duplicates and are representative of at least three independent experiments.

Phagocytosis of L. pneumophila by *Dictyostelium* was quantified by flow cytometry using bacteria constitutively expressing *gfp.* Approximately ten times more amoebae showed increased fluorescence if infected with wild-type L. pneumophila compared to an *icmT* mutant strain (Δ*icmT*), which lacks a functional Icm/Dot T4SS (Figure 1). This result indicates that at least ten times more wild-type L. pneumophila were phagocytosed compared to Δ*icmT.* Icm/Dot–dependent phagocytosis was observed at a multiplicity of infection (MOI) ranging from 1 to 100 and blocked by inhibitors of actin polymerization (latrunculin B, 20 μM; cytochalasin A, 10 μM), or by performing the infection at 4 °C.

**Figure 2:**
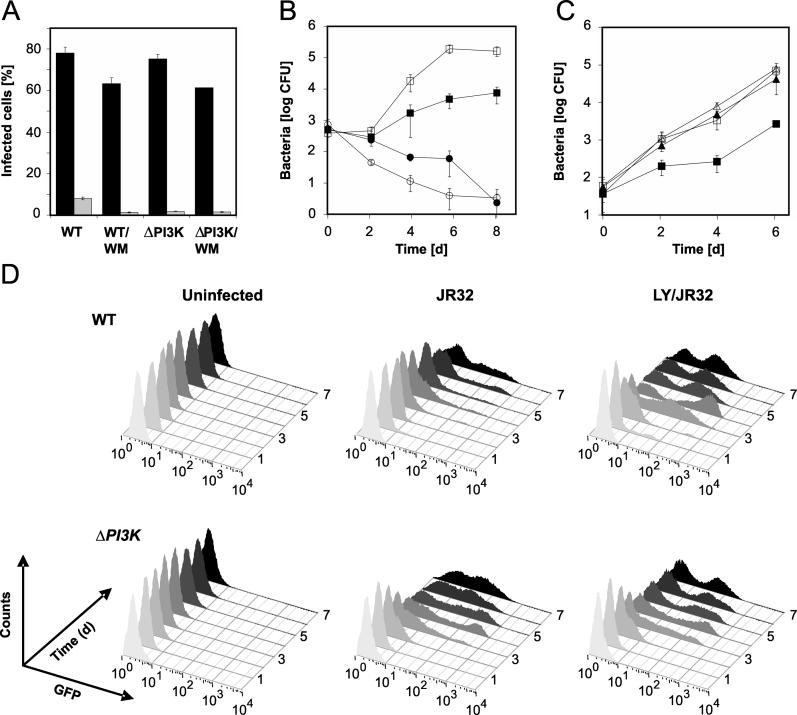
A Role for PI3Ks in Intracellular Replication but Not Phagocytosis of Wild-Type L. pneumophila (A) Phagocytosis by *Dictyostelium* wild-type Ax3 or Δ*PI3K1/2* (untreated or treated with 5 μM WM) of GFP-labeled L. pneumophila wild-type (black bars) or a Δ*icmT* mutant strain (grey bars) was determined by flow cytometry. (B) Release of *L. pneumophila* wild-type (squares) or Δ*icmT* (circles) into the supernatant of *Dictyostelium* wild-type (denoted by filled symbols) or Δ*PI3K1/2* (denoted by open symbols) was quantified by CFUs. (C) Release of wild-type *L. pneumophila* (CFUs) from *Dictyostelium* wild-type (filled squares denote untreated; filled triangles denote 10 μM LY) or Δ*PI3K1/2* (open squares denote untreated; open triangles denote LY). (D) Quantification by flow cytometry of intracellular growth of GFP-labeled wild-type L. pneumophila within wild-type *Dictyostelium* or Δ*PI3K1/2* in the presence or absence of 20 μM LY. The data shown are means and standard deviations of duplicates (A) or triplicates (B and C), and are representative of at least three independent experiments (A–D).

Wild-type L. pneumophila was only slightly less efficiently phagocytosed by *Dictyostelium* Δ*PI3K1/2* (−4%), or by wild-type *Dictyostelium* treated with the PI3K inhibitors wortmannin (WM, −19%) or LY294002 (LY, −33%), respectively (Figures 2A and S1A). Thus, genetic and pharmacological data indicate that phagocytosis of L. pneumophila by *Dictyostelium* does not require PI3Ks. This result is in agreement with the finding that the uptake of L. pneumophila by macrophage-like cells occurs via a WM-insensitive pathway [36]. Contrarily, phagocytosis of Δ*icmT* was reduced by 77%–88% upon deletion or inhibition of PI3Ks, corresponding to reports that *Dictyostelium* PI3K1 and PI3K2 are involved in phagocytosis of E. coli [37]. The addition of PI3K inhibitors to Δ*PI3K1/2* did not further diminish phagocytosis of Δ*icmT,* suggesting that other *Dictyostelium* class I PI3Ks present in the genome [38] are not involved in uptake.

### PI3Ks Are Involved in Intracellular Replication of Wild-Type L. pneumophila and Degradation of Δ*icmT*

The effect of PI3Ks on intracellular replication of L. pneumophila was quantified by determining colony-forming units (CFUs) released from lysed *Dictyostelium* into the supernatant of infected cultures. Compared to wild-type *Dictyostelium,* a factor of approximately 100 more wild-type L. pneumophila were released within 6–8 d from Δ*PI3K1/2* (Figure 2B) or amoebae treated with LY (Figure 2C), indicating that functional PI3Ks restrict intracellular replication of *L. pneumophila.* To test intracellular growth of L. pneumophila more directly, we analyzed *Dictyostelium* infected with green fluorescent protein (GFP)–labeled L. pneumophila by flow cytometry (Figure 2D). In this assay, GFP-labeled L. pneumophila grew earlier and more efficiently within Δ*PI3K1/2* or wild-type *Dictyostelium* treated with LY compared to untreated wild-type amoebae. Treatment of Δ*PI3K1/2* with LY did not enhance intracellular replication further, suggesting that no other class I PI3K is involved. Quantification by flow cytometry of GFP-labeled wild-type L. pneumophila released from *Dictyostelium* showed that L. pneumophila emerged earlier from *Dictyostelium* lacking PI3Ks, yet apparently grew at similar rates (Figure S1B). In a “single round” growth assay, where the amoebae were selectively lysed with saponin, L. pneumophila started to grow after just 1 d in the absence of PI3Ks, while at the same time in wild-type *Dictyostelium* the numbers of wild-type L. pneumophila still decreased (Figure S1C).

**Figure 3:**
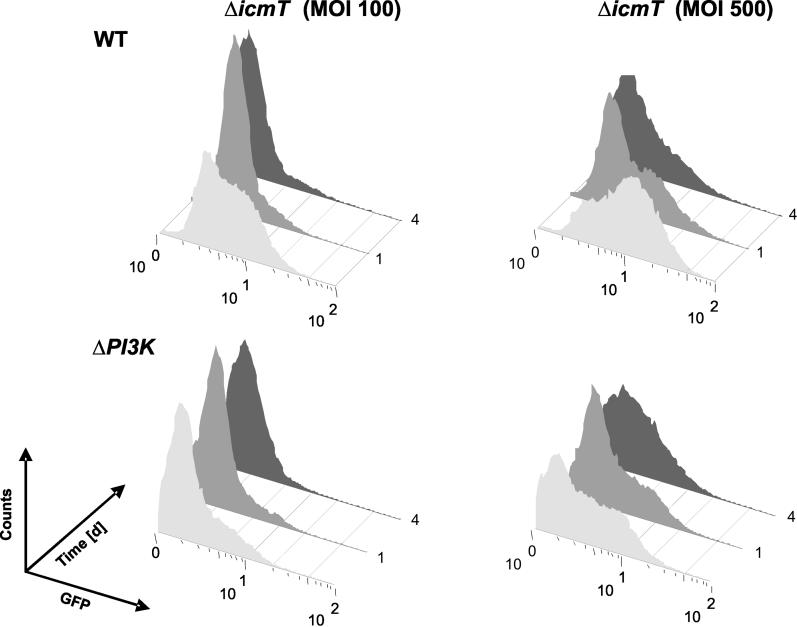
A Role for PI3Ks in Degradation of L. pneumophila Δ*icmT* Quantification by flow cytometry of intracellular degradation of GFP-labeled L. pneumophila Δ*icmT* (MOI 100, MOI 500) within wild-type *Dictyostelium* or Δ*PI3K1/2.* In the absence of PI3Ks, Δ*icmT* is less efficiently phagocytosed and degraded.

While Δ*icmT* did not replicate within *Dictyostelium* in the presence or absence of PI3Ks (Figure 2B), the mutant bacteria were killed approximately twice more slowly within Δ*PI3K1/2* (Figure 3; Table S1). These results are in agreement with a requirement of PI3Ks for the endocytic degradative pathway [24].

We also tested the effects of PI3K inhibitors on intracellular replication of L. pneumophila within macrophage-like cell lines. Treatment with 1 μM WM or 25 μM LY had no effect or slightly (3- to 5-fold) decreased the number of L. pneumophila released from murine RAW 264.7 cells or from differentiated human HL-60 macrophage-like cells (unpublished data).

### Trafficking of L. pneumophila Is Altered in the Absence of Functional PI3Ks

The finding that L. pneumophila replicates more efficiently in the absence of PI3Ks suggests that vesicle trafficking and formation of the LCV are altered. As a marker for LCVs, we used the ER membrane protein calnexin fused to GFP, which within 2 h co-localizes with about 65% LCVs harboring wild-type L. pneumophila but not at all with Δ*icmT*-containing LCVs (unpublished data; [6,9]). Calnexin does not profoundly affect trafficking of *L. pneumophila,* since intracellular replication within wild-type *Dictyostelium* was similar to replication in *Dictyostelium* mutants lacking calnexin, calreticulin, calnexin/calreticulin (Figure S2A), or *Dictyostelium* expressing calnexin-GFP (Figure S2B).

**Figure 4:**
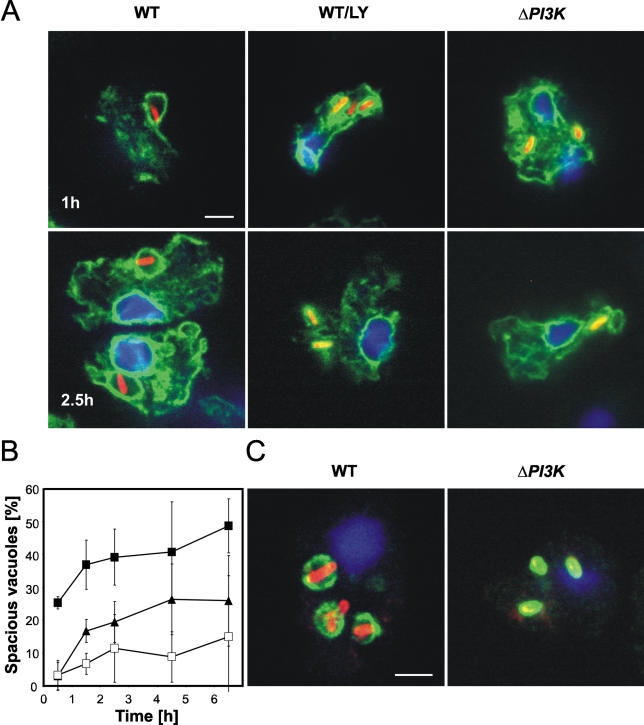
Trafficking of L. pneumophila within *Dictyostelium* Lacking Functional PI3Ks (A) Confocal laser scanning micrographs of calnexin-GFP–labeled *Dictyostelium* (green) wild-type Ax3 (WT denotes untreated or treated with 20 μM LY) or Δ*PI3K1/2* (denoted by ΔPI3K) infected with DsRed-Express–labeled wild-type L. pneumophila (red) for 1 h or 2.5 h, respectively. Representative examples of spacious vacuoles (WT) and tight vacuoles (WT/LY, ΔPI3K) are shown. DNA was stained with DAPI (blue). Bar denotes 2 μm. (B) Quantification over time of spacious LCVs in calnexin-GFP–labeled *Dictyostelium* wild-type (filled squares denote untreated; filled triangles denote LY) or Δ*PI3K1/2* (open squares) infected with DsRed-Express–labeled wild-type *L. pneumophila.* Data represent means and standard deviations of the percentage of spacious vacuoles from 50–200 total vacuoles per time point scored in four independent experiments. (C) Confocal laser scanning micrographs of *Dictyostelium* wild-type or Δ*PI3K1/2*, infected for 75 min with wild-type L. pneumophila expressing M45-tagged SidC. Infected amoebae were stained with rhodamine-conjugated anti-L. pneumophila antibody (red), FITC-conjugated anti-M45-tag antibody (green), and DAPI (blue), respectively. Bar denotes 2 μm.

In *Dictyostelium* wild-type and in strains lacking PI3Ks, the LCVs acquired calnexin-GFP with similar kinetics (unpublished data), suggesting that initial docking and fusion of ER-derived vesicles with the *Legionella* phagosome is not affected by PI3Ks. However, the morphological dynamic of the LCV was altered, as the transition from “tight” to “spacious” vacuoles was severely impaired in *Dictyostelium* lacking functional PI3Ks (Figure 4A). In wild-type *Dictyostelium,* 25% of the LCVs appeared spacious as early as 15 min post-infection and, within 2 h, 40% spacious vacuoles were scored (Figure 4B). Contrarily, in *Dictyostelium* lacking PI3Ks, the portion of spacious LCVs was less than 5% at 15 min post-infection, reached only 10% (Δ*PI3K1/2*) or 20% (LY-treated wild-type *Dictyostelium*) within 2 h, and remained below the level observed in wild-type *Dictyostelium* throughout the 6-h observation period. At later time points, the morphological assessment of the vacuoles became difficult, since infected *Dictyostelium* easily detached from the substratum, and LCVs harboring replicating bacteria appeared spacious in the presence or absence of PI3Ks. In summary, these results indicate that class I PI3Ks play a role in the dynamic modulation of the LCV and the formation of a replication-permissive vacuole.

### The Icm/Dot–Secreted L. pneumophila Protein SidC Localizes to Tight and Spacious Vacuoles

To correlate the morphology of the LCV with the presence of a putative L. pneumophila effector protein, we stained for the Icm/Dot–secreted protein SidC (*S*ubstrate of *I*cm/*D*ot transporter [18]). The function of SidC is unknown. However, the protein localizes to LCVs in *Legionella*-infected macrophages and is exposed to the cytoplasmic side of the vacuolar membrane. Immuno-staining of M45-tagged SidC within *Legionella*-infected *Dictyostelium* amoebae revealed its presence on spacious as well as tight LCVs (Figure 4C). Similar to LCVs labeled with calnexin-GFP, the majority of M45-SidC–labeled LCVs formed in wild-type *Dictyostelium* after 75 min appeared spacious, while at the same time the LCVs in Δ*PI3K1/2* were all tight-fitting (unpublished data). Some punctate background staining was also visible in uninfected *Dictyostelium* and thus is not due to association of SidC with cellular organelles. As even upon overexpression, M45-tagged SidC localized exclusively to the LCV, but not to other cellular vesicles, SidC anchors with high affinity and specificity to the LCV membrane.

In spacious vacuoles, L. pneumophila was frequently found to attach to the membrane of the LCV via its pole(s) (Figure 4A and 4C). Moreover, Icm/Dot substrates such as SidC, LidA, and the SidE family members have been reported to localize after secretion near the poles of L. pneumophila [17,18,39]. These findings suggest that L. pneumophila connects to the LCV membrane via Icm/Dot secretion system(s) localizing to the bacterial poles.

### SidC and SdcA Directly and Specifically Bind to PI(4)P In Vitro

**Figure 5:**
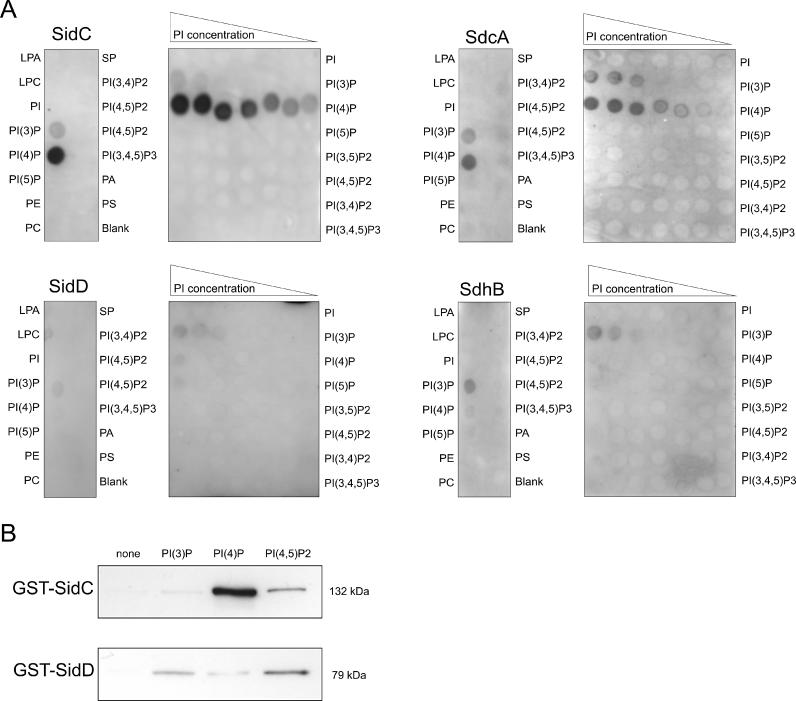
Binding of L. pneumophila Icm/Dot–Secreted Proteins to PIs In Vitro (A) Binding of affinity-purified GST fusion proteins of SidC, SdcA, SidD, or SdhB (160 pmol) to different lipids (100 pmol; left panels) or 2-fold serial dilutions of PIs (100–1.56 pmol; right panels) immobilized on nitrocellulose membranes was analyzed by a protein-lipid overlay assay using an anti-GST antibody. Lysophosphatidic acid is denoted by LPA; lysophosphocholine is denoted by LPC; sphingosine-1-phosphate is denoted by SP; phosphatidic acid is denoted by PA; and phosphatidylserine is denoted by PS. The experiment was reproduced at least three times with similar results. (B) PL vesicles (20 μl, 1 mM lipid) composed of PC (65%), PE (30%), and 5% (1 nmol) either PI(4)P, PI(3)P or PI(4,5)P_2_ were incubated with affinity-purified GST-SidC or GST-SidD (40 pmol), centrifuged, and washed. Binding of GST fusion proteins to PL vesicles was assayed by Western blot with an anti-GST antibody. Similar results were obtained in three separate experiments.

Intracellular replication of L. pneumophila depends on PI metabolism, as well as on the Icm/Dot T4SS. A direct link between these host cell and pathogen factors would exist if secreted L. pneumophila proteins bind to PIs on the LCV. SidC is an attractive candidate to test this hypothesis, since the protein binds to the LCV membrane, yet no transmembrane helices are predicted from its primary sequence. To determine whether SidC interacts with PIs in vitro, we assayed binding of an N-terminal GST-SidC fusion protein to PIs and other lipids immobilized on nitrocellulose membranes. Under these conditions, SidC directly and almost exclusively bound to PI(4)P and, to a much weaker extent, to PI(3)P but not to other PIs or lipids (Figure 5A). Estimated from binding of SidC to PIs arrayed in 2-fold serial dilutions, the affinity of SidC for PI(4)P was a factor of 50–100 higher than for PI(3)P. The SidC paralog SdcA (72% identity on an amino acid level) also specifically bound to PI(4)P and, to a lesser extent, to PI(3)P; yet compared with SidC, SdcA bound with apparently lower affinity to PI(4)P and higher affinity to PI(3)P.

We also tested GST fusion proteins of SidD and SdhB for binding to PIs (Figure 5A). SdhB is a paralog of SidH [18] and is predicted with low stringency by the “scansite” algorithm (http://scansite.mit.edu) to contain an ANTH domain putatively binding PI(4,5)P_2_. While the GST-SidD fusion protein did not bind to any of the lipids tested in vitro, the GST-SdhB fusion protein bound very weakly only to PI(3)P but not to other lipids.

To investigate the binding specificity of SidC to PIs incorporated into phospholipid (PL) vesicles, we incubated GST-SidC with PL vesicles composed of phosphatidylcholine (PC, 65%), phosphatidylethanolamine (30%), and 5% either PI(4)P, PI(3)P, or PI(4,5)P_2_. GST-SidD was used as a putative negative control. The PL vesicles were incubated with GST-SidC or GST-SidD, centrifuged, and washed several times prior to analyzing binding of the GST fusion proteins by Western blot with an anti-GST antibody (Figure 5B). Under the conditions used, SidC almost exclusively bound to PI(4)P, while binding to PI(3)P was negligible. Binding of SidC to PI(4,5)P_2_ was in the range observed for SidD and thus was considered unspecific. Estimated by densitometry, about 200 times more SidC bound to PL vesicles harboring PI(4)P compared to vesicles containing PI(3)P or PI(4,5)P_2_. Taken together, using two different biochemical assays, SidC was found to specifically bind to PI(4)P in vitro.

### SidC Preferentially Binds to LCVs in the Absence of Functional PI3Ks

**Figure 6:**
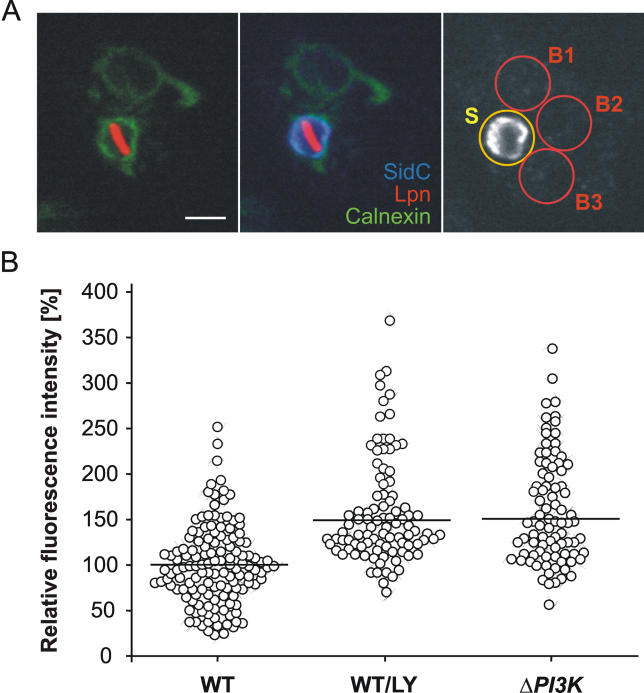
PI3Ks Affect the Amount of SidC Bound to LCVs in *Dictyostelium* (A) Confocal laser scanning micrographs of calnexin-GFP–labeled *Dictyostelium* wild-type strain Ax3 (green), infected with DsRed-Express–labeled wild-type L. pneumophila (red) for 1 h (left panel), and immuno-labeled for SidC (blue) with an affinity-purified primary and Cy5-conjugated secondary antibody (middle panel). To quantify fluorescence intensity (right panel), the averaged fluorescence intensity of background areas (B1, B2, and B3) was subtracted from the intensity of the sample area (S). Bar denotes 2 μm. (B) Dot plot of SidC fluorescence (average and variance) on LCVs within *Dictyostelium* wild-type (untreated, *n* = 135; 20 μM LY, *n* = 94) or Δ*PI3K1/2* (*n* = 86). The data shown are combined from six independent experiments, each normalized to 100% (average SidC fluorescence on LCVs in wild-type *Dictyostelium*).

To address the question of whether SidC binds to PI(4)P on LCVs in infected *Dictyostelium* amoebae, we analyzed whether altering the ratio of cellular PIs affects the amount of SidC bound to LCVs. In *Dictyostelium* Δ*PI3K1/2,* the level of the PI3K products PI(3,4)P_2_ and PI(3,4,5)P_3_ is decreased, while the level of the PI3K substrate PI(4)P is increased compared to the complemented strain [37]. Accordingly, if the level of PIs on LCVs mirrors the cellular levels of PI, SidC is predicted to preferentially bind to LCVs in the absence of PI3Ks. To test whether PI3Ks affect the amount of SidC on LCVs, SidC bound to LCVs was quantified by immunofluorescence using an affinity-purified antibody (Figure 6A). SidC and calnexin-GFP always and strictly co-localized on LCVs regardless of whether PI3Ks were present or not. However, we found that with high statistical significance (*p* < 10^−9^), approximately a factor of 1.5 more SidC localized to LCV membranes in Δ*PI3K1/2* or in wild-type *Dictyostelium* treated with LY, compared to LCVs formed in wild-type *Dictyostelium* (Figure 6B). This result is in agreement with the notion that, in the absence of functional PI3Ks, the amount of cellular and vacuolar PI(4)P is increased, allowing more SidC to bind to the LCV in L. pneumophila-infected host cells.

### PI(4)P Is a Lipid Marker of LCVs Harboring Icm/Dot–Proficient L. pneumophila

**Figure 7:**
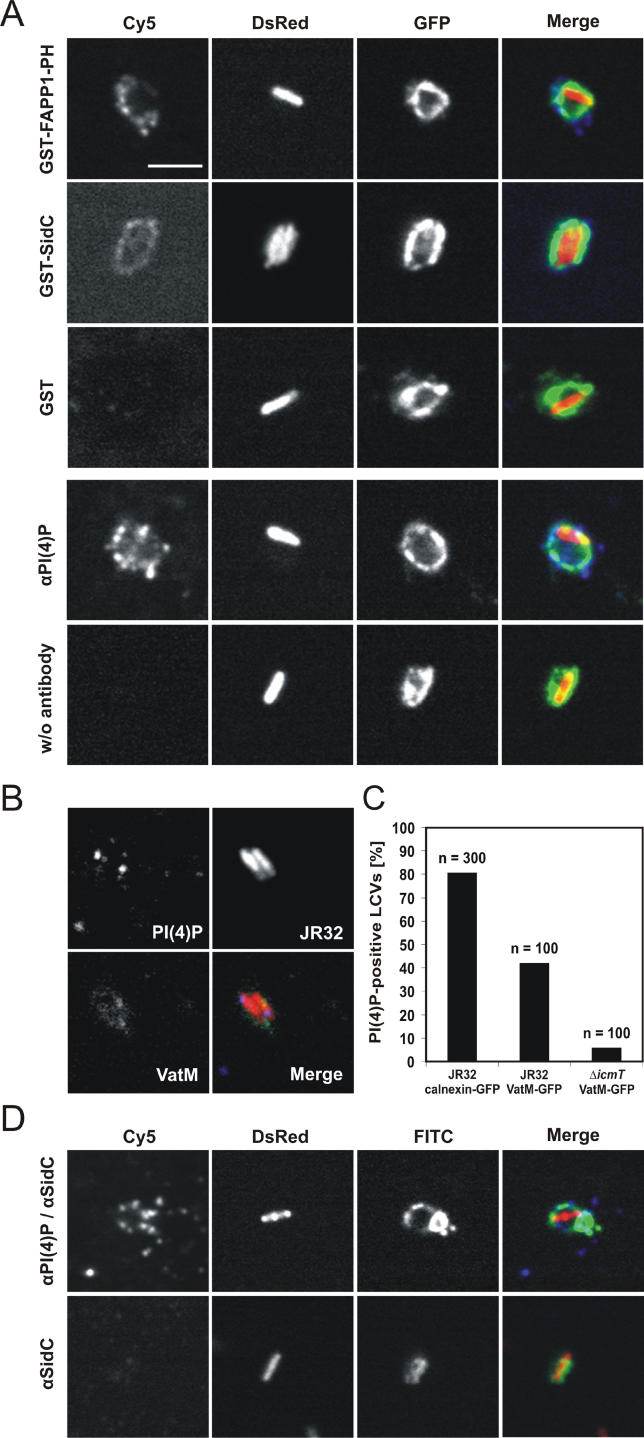
PI(4)P Is a Lipid Marker of LCVs Harboring Icm/Dot–Proficient L. pneumophila (A, B, and D) Confocal micrographs of LCVs in lysates of (A) calnexin-GFP–labeled *Dictyostelium,* (B) VatM-GFP–labeled *Dictyostelium,* or (D) RAW264.7 macrophages infected with DsRed-Express–labeled L. pneumophila are shown. The lysates were prepared with a ball homogenizer, and PI(4)P was visualized on the LCVs using as probes either the PH domain of the PI(4)P-binding protein FAPP1 fused to GST, an antibody against PI(4)P, or GST-SidC. Using GST alone or omission of the anti-PI(4)P antibody did not label the LCVs. Bar denotes 2 μm (magnification of all images is identical). (C) Quantification of PI(4)P-positive calnexin-GFP–labeled (*n* = 300) or VatM-GFP–labeled (*n* = 100) LCVs in *Dictyostelium* wild-type strain Ax3.

SidC specifically binds to PI(4)P in vitro and to the membrane of the LCV, suggesting that PI(4)P is a constituent of the LCV. To test whether PI(4)P is indeed a lipid marker of the LCV, we used as probes a PI(4)P-specific antibody or the PH domain of FAPP1 (phosphatidylinositol(4) phosphate adaptor protein-1) fused to GST. FAPP1 is required for transport from the *trans* Golgi network to the plasma membrane and has been shown to specifically bind PI(4)P [40,41]. The PI(4)P-specific antibody, as well as the GST-FAPP1-PH probe, labeled calnexin-GFP–positive LCVs in homogenates of *Dictyostelium* infected with L. pneumophila (Figure 7A). Similarly, GST-SidC stained the LCV in homogenates of L. pneumophila-infected *Dictyostelium*. Using the PI(4)P-specific antibody, we found that 80% of calnexin-GFP–positive, wild-type L. pneumophila-containing vacuoles stain positive for PI(4)P. Omission of the anti-PI(4)P antibody or using GST alone did not label the LCV. These results establish PI(4)P as a lipid marker of the LCV in L. pneumophila-infected *Dictyostelium*. In intact calnexin-GFP–labeled *Dictyostelium* infected with *L. pneumophila,* the PI(4)P probes produced a punctate staining on the cytoplasmic membrane and in the cytoplasm, rendering it difficult to detect PI(4)P on the LCVs (unpublished data).

To address the question of whether the presence of PI(4)P on LCVs is dependent on the Icm/Dot T4SS, we used the *Dictyostelium* wild-type strain Ax3 expressing VatM-GFP. VatM is the 100-kDa transmembrane subunit of the vacuolar H^+^-translocating adenosine triphosphatase (V-ATPase), which is excluded from LCVs harboring wild-type L. pneumophila but is delivered to LCVs containing Δ*icmT* by fusion with endolysosomes [9,19]. One hour post-infection, only 15% of wild-type but 41% of Δ*icmT* mutant *L. pneumophila* resided in vacuoles staining positive for VatM-GFP. Interestingly, however, 42% of the VatM-GFP–positive LCVs harboring wild-type L. pneumophila stained positive for PI(4)P, compared to only 6% of VatM-positive LCVs containing Δ*icmT* (Figure 7B and 7C). These results indicate that the presence of PI(4)P on LCVs is Icm/Dot–dependent.

The mechanism of intracellular replication of L. pneumophila within amoebae and macrophages appears to be very similar. To test whether LCVs formed in macrophages also contain PI(4)P, we used RAW264.7 cells. L. pneumophila grows within these macrophages [1,42] and, therefore, the corresponding LCVs represent replication-permissive compartments. In lysates of RAW264.7 macrophages infected with *L. pneumophila,* the LCVs were labeled by an anti-PI(4)P antibody as well as by an anti-SidC antibody (Figure 7D). As expected, upon omission of the anti-PI(4)P antibody, only SidC was detected on the LCV. These results demonstrate that PI(4)P is also a lipid component of the LCV in macrophages, and the results further underscore the structural similarity of LCVs within amoebae and macrophages. Similar to *Dictyostelium,* in intact L. pneumophila-infected macrophages, the PI(4)P probes led to a punctate staining pattern on the cytoplasmic membrane and in the cytoplasm (unpublished data).

## Discussion

The Icm/Dot T4SS is well established as a pivotal virulence determinant of *L. pneumophila,* which governs phagocytosis as well as intracellular trafficking of the bacteria. Contrarily, the activities and host cell targets of most of the Icm/Dot–secreted effector proteins remain obscure. Here, we analyze the role of host cell PI3Ks during phagocytosis and intracellular replication of L. pneumophila and identify Icm/Dot–secreted proteins that directly engage PI(4)P.

Wild-type *L. pneumophila* upregulates phagocytosis by *Dictyostelium* (Figures 1 and 2) and by macrophages or Acanthamoeba castellanii [14]. PI3Ks were found to be dispensable for phagocytosis of wild-type L. pneumophila by *Dictyostelium* but were found to be involved in phagocytosis and degradation of an L. pneumophila Δ*icmT* mutant. Therefore, L. pneumophila apparently employs a specific phagocytic pathway, which bypasses a requirement for PI3Ks. This pathway is distinct from PI3K-dependent phagocytosis of non-invasive or other pathogenic bacteria, including Listeria monocytogenes or uropathogenic E. coli [26].

We provided genetic and pharmacological evidence that class I PI3Ks are involved in intracellular replication and trafficking of wild-type L. pneumophila in *Dictyostelium* (Figures 2 and 4). In the absence of PI3Ks, L. pneumophila replicated more efficiently and, at the same time, the transition from tight to spacious vacuoles was inhibited. Contrarily, in a *Dictyostelium rtoA* mutant, the defective transition of LCVs from tight to spacious vacuoles coincided with a decreased efficiency of intracellular replication of L. pneumophila [8]. Our results suggest that the “maturation” of tight to spacious vacuoles is not required for formation of a replication-permissive vacuole. PI3Ks have been implicated in homotypic phagosome fusion and formation of spacious phagosomes [43]. Accordingly, PI3K-dependent formation of spacious phagosomes might represent a host cell process which does not support (or which even counteracts) the formation of a replication-permissive LCV.

Formation of the LCV takes place at ER exit sites and requires the evasion of the endosomal pathway and a functional early secretory pathway [5–7]. Since class I PI3Ks play a role in endosomal degradation of Δ*icmT* (Figure 3) and the modulation of the LCV (Figure 4A and 4B), an absence of PI3Ks might contribute to a more efficient intracellular replication of L. pneumophila in two synergistic ways: (i) by rendering the degradative endocytic pathway less efficient, and (ii) by promoting interactions of the LCV with the secretory pathway. The PI3K products PI(3,4,5)P_3_ and PI(3)P have been shown to promote phagocytosis, endocytosis, and bacterial degradation [24]. An absence of these PIs might therefore account for the observed defects in degradation of Δ*icmT* and render evasion of the degradative pathway by wild-type L. pneumophila more efficient. On the other hand, the effect of PI3Ks on trafficking of LCVs along the secretory pathway perhaps involves PI(4)P, which in the absence of PI3Ks is more abundant in *Dictyostelium* [37] and thus might accumulate locally on LCVs. The discovery that the Icm/Dot–secreted proteins SidC and SdcA bind PI(4)P in vitro (Figure 5) and anchor to the LCV in infected *Dictyostelium* preferentially in the absence of PI3Ks (Figure 6) supports this hypothesis.

To account for their effect on the morphological dynamics of LCVs, PI3Ks might be recruited and act in *cis,* or the absence of PI3Ks might affect the modulation of LCVs in *trans* by increasing the cellular concentration of PI(4)P. The mechanism regulating PIs on the LCV is expected to be complex and likely involves other PI-metabolizing host cell enzymes, as well as additional Icm/Dot–secreted bacterial proteins. The finding that PI3K inhibitors assist intracellular replication of L. pneumophila in *Dictyostelium,* but not in macrophages, suggests that in protozoan and metazoan cells PI3Ks are inhibited with different efficiencies and is consistent with the hypothesis that other PI kinases are also involved in the process. It is noteworthy that the PI3K inhibitors WM and LY also inhibit type III PI4Ks [44]. PI3Ks and PI4Ks are expected to affect the amount of PI(4)P on LCVs in opposite ways, providing a possible explanation for the apparently inconsistent results obtained with the pharmacological inhibitors. In any case, the mechanism by which L. pneumophila subverts host cell PI metabolism is probably conserved in amoebae and mammalian cells, since LCVs in either host cell harbor PI(4)P (Figure 7).

The identification of Icm/Dot–secreted L. pneumophila proteins specifically binding to PI(4)P suggested that this PI is a constituent of the LCV. Using an anti-PI(4)P antibody or a GST-FAPP1-PH probe, we could directly confirm that PI(4)P is a lipid marker of LCVs. PI(4)P accumulates in the *trans* Golgi complex and regulates exocytosis by a poorly defined mechanism [25]. Thus, PI(4)P is the first Golgi marker and the first lipid marker identified on the LCV. SidC secreted by L. pneumophila selectively bound to the LCV, but not to other cellular vesicles, even if overexpressed as an M45-tagged protein (Figures 4C and 6). To account for this specificity, SidC perhaps engages a co-receptor on the LCV, which might further increase the affinity of the protein to PI(4)P. However, SidC alone also binds to PI(4)P, as demonstrated with purified GST-SidC in vitro (Figure 5).

SidC and SdcA do not harbor any obvious catalytic domain. However, both proteins contain extended regions predicted to form coiled-coils, suggesting that these proteins engage in protein–protein interactions. By anchoring to PI(4)P, SidC and SdcA either (i) directly engage in vesicle trafficking and the formation of a replicative vacuole via an effector domain, or (ii) serve as adaptors for other L. pneumophila effectors involved in formation of the replicative vacuole or in the exit of the bacteria from host cells. SidC and its upstream paralog SdcA have no orthologs in the database, yet are found in the genomes of all three L. pneumophila strains (Philadelphia, Paris, and Lens) sequenced to date [45,46]. The proteins are not predicted to contain a PH or other PI-binding domains, and therefore, likely harbor a novel PI(4)P-binding domain. Owing to its high degree of specificity, the PI(4)P-binding domain of SidC/SdcA might serve as a selective PI(4)P probe in biochemical and cell biological assays.

## Materials and Methods

### Growth of bacteria, *Dictyostelium,* and macrophages.

The L. pneumophila strains used in this study were wild-type strain JR32 (a salt-sensitive derivative of a streptomycin-resistant Philadelphia-1 strain), the isogenic Δ*icmT* deletion mutant GS3011, which lacks a functional Icm/Dot T4SS, and corresponding strains constitutively producing enhanced GFP or the red fluorescent protein DsRed-Express (Table 1). L. pneumophila was routinely grown for 3 d on charcoal yeast extract (CYE) agar plates, buffered with *N*-(2-acetamido)-2-aminoethane-sulfonic acid [47]. Liquid cultures were inoculated in AYE medium supplemented with BSA (0.5%) [48] at an OD_600_ of 0.1 and grown for 21 h at 37 °C (post–exponential growth phase). To maintain plasmids, chloramphenicol (cam) was added at 5 μg/ml. As “input” controls, 20 μl of a 10^5^/ml bacterial solution was plated and counted after 3 d incubation in all phagocytosis and intracellular growth experiments.

**Table 1:**
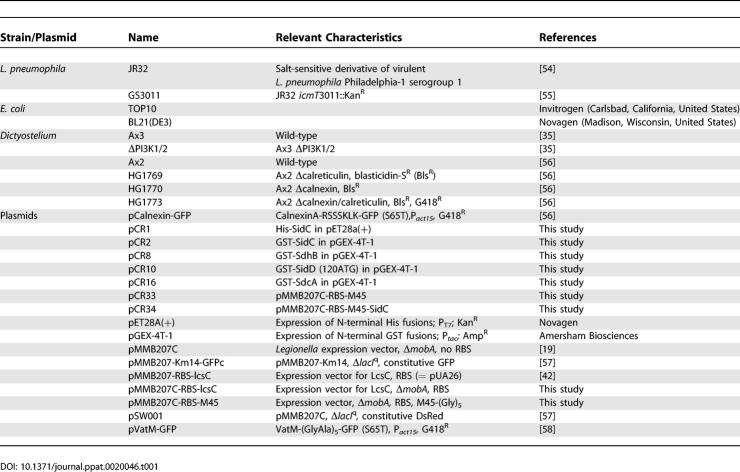
Strains and Plasmids.

D. discoideum wild-type strain Ax3 and the *PI3K1/2* double mutant (Δ*PI3K1/2*) were a gift from R. Firtel (University of California San Diego, San Diego, California, United States). The Δ*PI3K1/2* mutant is lacking two PI3Ks that are related to the mammalian p110 catalytic subunit of class I PI3Ks. The mutant strain shows morphological, developmental, and chemotactic phenotypes and is defective for vegetative growth in axenic medium and on bacterial lawns [35,37,43,49]. Specifically, Δ*PI3K1/2* is smaller than the isogenic wild-type strain and is impaired for (i) phagocytosis of live or autoclaved bacteria, (ii) pinocytosis of fluid markers, (iii) maturation of phagosomes to “spacious” phagosomes via homotypic fusion, and (iv) possibly exocytosis. A biochemical analysis of the PI profile of Δ*PI3K1/2* compared to the complemented strain revealed that the levels of PI(3,4)P_2_ and PI(3,4,5)P_3_ were reduced while the level of PI(4)P was elevated, and that PI(3)P, as well as PI(4,5)P_2_, remained unchanged [37].

*Dictyostelium* amoebae were grown axenically at 23 °C in 75 cm^2^ tissue culture flasks in HL5 liquid medium (10 g of glucose, 5 g of yeast extract, 5 g of proteose peptone, 5 g of thiotone E peptone, 2.5 mM Na_2_HPO_4_, 2.5 mM KH_2_PO_4_ in 1 l of H_2_O [pH 6.5]), supplemented with 10 μg/ml of G418 or blasticidin-S when necessary. The amoebae were split once or twice a week and fed with fresh HL5 medium 24 h before use. For viability assays, *Dictyostelium* was plated together with Klebsiella pneumoniae on SM/5 agar plates, and plaque-forming units (PFU) were counted after 3–4 d incubation at 23 °C [50].

Murine RAW264.7 macrophages and human HL-60 cells were cultivated in RPMI1640 medium supplemented with 10% FCS and 2 mM l-glutamine at 37 °C in a humidified atmosphere of 5% CO_2_. The HL-60 cells were differentiated into macrophage-like cells by incubation for 2 d with 100 ng/ml of phorbol 12-myristate 13-acetate.

### Plasmid construction, protein purification, and antibody preparation.

Translational *gst* fusions of *sidC, sdcA, sidD,* and *sdhB,* were constructed by PCR amplification of the putative open reading frames (ORFs) using the primers listed in Table S2. For *sidD,* the ATG at position 120 downstream of a TTG in the ORF was used as a start codon. The PCR fragments were cut with BamHI and SalI and ligated into plasmid pGEX-4T-1 yielding pCR2, pCR16, pCR10, and pCR8, respectively (Table 1). All constructs were sequenced. Production of the fusion proteins in E. coli BL21(DE3) was induced at a cell density (OD_600_) of 0.6 with 0.5 mM isopropyl-1-thio-β-d-galactopyranoside (IPTG) for 3 h at 30 °C in LB medium. In all cases, this protocol resulted in a significant portion of soluble fusion protein of the expected size (132 kDa, 132 kDa, 79 kDa, and 239 kDa, respectively). The fusion proteins were purified from lysates prepared by sonication using glutathione-sepharose beads in a batch procedure according to the manufacturer's recommendations (Amersham Biosciences, Little Chalfont, United Kingdom). Purity of the protein preparations was analyzed by SDS polyacrylamide gel electrophoresis.

A translational *his*-*sidC* fusion was constructed by moving the *sidC* ORF (cut with BamHI and SalI) from pCR2 into pET28a(+), yielding pCR1. Production of His_6_-SidC by E. coli BL21(DE3) was induced with 1 mM IPTG for 3–6 h at 30 °C, resulting in a predominantly soluble protein of the expected size (109 kDa). The His_6_-SidC fusion protein was purified by Ni^2+^ affinity chromatography, and polyclonal antibodies against the purified protein were raised in rabbits (NeoMPS). The antibodies were affinity-purified from rabbit serum using an Aekta liquid chromatography system (Amersham Biosciences) and GST-SidC covalently linked to Affigel-10 beads (BioRad, Hercules, California, United States) [51]. Typical yield of purified anti-SidC antibody was 2.2–3.2 mg/ml serum.

An L. pneumophila expression vector for M45-tagged SidC (pCR34) was constructed using pMMB207C [19] as a backbone. First, a ribosomal binding site (RBS) was introduced into pMMB207C by moving an EcoRI/BamHI fragment from pUA26 [42], yielding pMMB207C-RBS-*lcsC.* To insert the DNA encoding the M45 tag, the oligonucleotides (oligos) oCR-P1 and oCR-P2 harboring a mutation in the internal BamHI site of the M45 sequence (G to T in oligo oCR-P1) were used. The oligos (1 nmol each in 100 μl) were annealed (heated to 94 °C, followed by slow cooling to 4 °C) and ligated into pMMB207C-RBS-*lcsC* cut with NdeI and BamHI, yielding vector pMMB207C-RBS-M45. Finally, the fragment encoding SidC was moved from plasmid pCR2 into pMMB207C-RBS-M45 by using the BamHI and SalI restriction sites, yielding pCR34.

### Analysis of phagocytosis by flow cytometry.

Phagocytosis of L. pneumophila by *Dictyostelium* was analyzed by flow cytometry using GFP-labeled bacteria. Exponentially growing *Dictyostelium* was seeded onto a 24-well plate (5 × 10^5^ cells/ml HL5 medium per well) and were allowed to adhere for 1–2 h. L. pneumophila grown for 21 h in AYE liquid culture was diluted in HL5 medium and used to infect the amoebae at an MOI of 100 or at the MOI indicated (OD_600_ of 0.3 = 2 × 10^9^ bacteria/ml). The infection was synchronized by centrifugation (10 min, 880 *g*), infected cells were incubated at 25 °C and, 30 min post-infection, extracellular bacteria were removed by washing three to five times with SorC (2 mM Na_2_HPO_4_, 15 mM KH_2_PO_4_, 50 μM CaCl_2_ [pH 6.0]). Infected *Dictyostelium* was detached by vigorously pipetting, and 2 × 10^4^ amoebae per sample were analyzed using a FACSCalibur flow cytometer (Becton Dickinson, Palo Alto, California, United States). The GFP fluorescence intensity falling into a *Dictyostelium* scatter gate was quantified using FlowJo software (Treestar, http://www.treestar.com).

To confirm that the fluorescence observed arises from internalized and not from adherent *L. pneumophila,* phagocytosis was inhibited in parallel experiments. One hour prior to infection, the medium was exchanged, and *Dictyostelium* was incubated in HL5 medium containing either latrunculin B (1–50 μM), cytochalasin A (1–50 μM) or, as a solvent control, DMSO (0.5%). Alternatively, the infection was performed on ice, and the infected cells were incubated at 4 °C. While latrunculin B or cytochalasin A blocked phagocytosis in a dose-dependent manner, cytochalasin D (up to 50 μM), which effectively blocks macrophage phagocytosis, did not prevent uptake of L. pneumophila by *Dictyostelium* (unpublished data). Moreover, latrunculin B did not affect binding of L. pneumophila to *Dictyostelium* (unpublished data).

In experiments addressing the role of PI3Ks, the amoebae were incubated in HL5 medium containing the PI3K inhibitors WM (0.1–10 μM) or LY (5–25 μM) for 1 h prior to infection, which was then performed in the presence of the inhibitors. At the concentrations indicated, the pharmacological inhibitors did not affect the viability of L. pneumophila or *Dictyostelium,* as determined by measuring CFU or PFU (unpublished data).

### Intracellular growth of L. pneumophila within *Dictyostelium.*

Release of L. pneumophila from *Dictyostelium* owing to intracellular replication was quantified by determining CFUs in the supernatant as described [32,34]. Briefly, exponentially growing *Dictyostelium* amoebae were washed with SorC and resuspended in MB medium (7 g of yeast extract, 14 g of thiotone E peptone, 20 mM MES in 1 l of H_2_O [pH 6.9]). *Dictyostelium* (1 × 10^5^ cells per well) was seeded onto a 96-well plate, allowed to adhere for 1–2 h, and infected at an MOI of 1 with L. pneumophila grown on CYE plates for 3–4 d and resuspended in MB medium. Occasionally, L. pneumophila grown in AYE medium for about 21 h was used as an inoculum. The infection was synchronized by centrifugation, and the infected amoebae were incubated at 25 °C. At the time points indicated, the number of bacteria released into the supernatant was quantified by plating aliquots (10–20 μl) of appropriate dilutions on CYE plates. L. pneumophila did not grow in MB medium. Rather, the CFUs decreased by two to three orders of magnitude within 3–6 d under these conditions (unpublished data).

Intracellular bacterial growth before host cell lysis was quantified by counting CFUs after selectively lysing infected *Dictyostelium* with saponin (“single-round replication”). At the time points indicated, the MB medium was replaced by 100 μl of 0.8% saponin and incubated for 15 min. The cells were lysed by pipetting, and aliquots were plated.

Intracellular replication of GFP-labeled wild-type L. pneumophila or killing of GFP-labeled Δ*icmT* was also directly determined by flow cytometry. Here, the fluorescence intensity falling into a *Dictyostelium* scatter gate was quantified. Alternatively, the number of GFP-labeled L. pneumophila released into 120 μl of *Dictyostelium* supernatant was quantified by flow cytometry using a scatter gate adjusted for bacteria.

To determine the effect of PI3K inhibitors on intracellular growth of *L. pneumophila, Dictyostelium* was incubated for 1 h in MB medium containing 5 μM WM or 10–20 μM LY, respectively. The medium was not exchanged prior to infection with *L. pneumophila,* leaving the inhibitors throughout the experiment. Since WM is unstable in buffered aqueous solutions [52], LY was used preferentially. In some experiments, the inhibitors were added freshly to the medium every second day of the incubation period, yet this protocol did not alter the results of the experiments. The PI3K inhibitors did not have an effect on L. pneumophila in MB medium (unpublished data). *Dictyostelium* Ax3 wild-type cells treated with 5 μM WM or 10 μM LY were as viable as untreated wild-type or Δ*PI3K1/2* for up to 5 or 6 d in MB medium (unpublished data). At later time points, cells treated with LY showed a reduced viability as determined by PFU on lawns of *K. pneumoniae,* and therefore, intracellular growth of L. pneumophila in the presence of PI3K inhibitors was analyzed for only up to 6 d.

### Intracellular trafficking of L. pneumophila and constituents of LCVs analyzed by immunofluorescence.

For immunofluorescence, *Dictyostelium* or macrophages were split and fed 2 d prior to an experiment, seeded on sterile coverslips in 24-well plates at 2.5 × 10^5^ per well in 0.5 ml of HL5 medium *(Dictyostelium)* or RPMI medium (macrophages), and allowed to grow overnight. The medium was exchanged about 1 h before the infection and contained 20 μM LY where indicated. The L. pneumophila strains used for the infections were grown for 21 h (OD_600_ of the inoculum: 0.1) in 3 ml of AYE/BSA containing 5 μg/ml of cam and 0.5 mM IPTG when required. Bacterial cultures were diluted in HL5 medium *(Dictyostelium)* or RPMI medium (macrophages) to a concentration of 5 × 10^8^/ml, and 100 μl of the suspension was added to the phagocytes (MOI = 100). The infection was synchronized by centrifugation, and the cells were washed twice with HL5 medium *(Dictyostelium)* or PBS (macrophages), respectively.

At the time points indicated, the infected phagocytes were washed three times with cold SorC buffer *(Dictyostelium)* or PBS (macrophages) and fixed with 4% paraformaldehyde for 30 min at 4 °C. The fixed cells were washed three times, permeabilized (0.1% Triton X-100, 10 min) and blocked with 2% normal human AB serum in SorC or PBS for 30 min. The coverslips were incubated for 1 h at room temperature on parafilm with 30 μl of primary antibodies diluted in blocking buffer (rhodamine-conjugated rabbit anti-L. pneumophila Philadelphia-1 serogroup 1, 1:100 [m-Tech, Monoclonal Technologies, http://www.4m-tech.com]; mouse anti-M45 hybridoma supernatant, 1:4 [53]; monoclonal mouse anti-GST 1: 200 [Sigma, St. Louis, Missouri, United States]; affinity-purified rabbit anti-SidC, 1:1000 [see above]; and mouse IgM anti-PI(4)P, 1: 200 [Echelon Biosciences, http://www.echelon-inc.com]) and washed three times with blocking buffer after each antibody. Secondary antibodies were from Jackson ImmunoResearch (West Grove, Pennsylvania, United States)—FITC-conjugated goat anti-mouse IgG; FITC-conjugated goat anti-rabbit IgG; Cy5-conjugated goat anti-mouse IgG and IgM; Cy5-conjugated goat anti-rabbit IgG—and incubated at a 1:200 dilution in blocking buffer for 1 h at room temperature. Finally, DNA was stained with DAPI (1 μg/ml) in SorC or PBS for 5 min, and the coverslips were washed twice and mounted using Vectashield (Vector Laboratories http://www.vectorlabs.com).

The amount of SidC on LCVs harboring DsRed-Express–labeled L. pneumophila in calnexin-GFP–labeled wild-type *Dictyostelium* (untreated or treated with 20 μM LY) or in Δ*PI3K1/2* was quantified by immunofluorescence using affinity-purified anti-SidC and Cy5-conjugated secondary antibodies. The fluorescence intensity of an area identical for all samples and covering the LCV was quantified using Quantity One software (BioRad) after background correction (with an averaged intensity of three areas within the infected amoeba). To standardize the procedure, all images were acquired with the same exposure time. Only LCVs containing rod-shaped and non-permeabilized bacteria were considered, and “equatorial” sections along the *z*-axis through the bacteria were chosen. “Spacious” vacuoles were defined as vacuoles where the calnexin-GFP–labeled membrane surrounding L. pneumophila was clearly detached, leaving a space between the membrane and the bacterium; all other LCVs observed were scored as “tight”.

PI(4)P was visualized in homogenates of infected *Dictyostelium* using as probes the affinity-purified GST-FAPP1-PH domain [40,41], GST-SidC, or an anti-PI(4)P antibody (Echelon Biosciences). Calnexin-GFP–labeled *Dictyostelium* (2 × 10^7^) was infected with DsRed-Express–labeled L. pneumophila (MOI 100), suspended in 3 ml of homogenization buffer (20 mM HEPES-KOH [pH 7.2], 250 mM sucrose, 0.5 mM EGTA [6]) and lysed by ten passages through a ball homogenizer (Isobiotech, http://www.isobiotec.com) using an exclusion size of 8 μm. The homogenate was immobilized by centrifugation (10 min, 850 *g*) onto coverslips coated with poly-l-lysine. Subsequently, the probes (GST-FAPP1-PH, GST-SidC, and GST, 4 μM each) were added for 15 min in the presence of 1 mM ATP, fixed (4% paraformaldehyde, 30 min), washed with SorC, and blocked (2% NHS in SorC, 30 min). Binding of the probes to the LCV was visualized as described above with an anti-GST antibody and a Cy5-conjugated secondary antibody. In experiments where the anti-PI(4)P antibody was used, the infected cells were fixed immediately after immobilization on poly-l-lysine–coated coverslips and otherwise processed as described above. PI(4)P on LCVs in RAW264.7 macrophages was detected with the anti-PI(4)P antibody. The infected macrophages were treated identically to *Dictyostelium* except that PBS was used as a buffer in all steps.

The samples were viewed with an inverted confocal microscope (Axiovert 200M; Zeiss, http://www.zeiss.com), equipped with a ×100 oil-phase contrast objective (Plan Neofluar; Zeiss), an “Ultraview” confocal head (PerkinElmer, Wellesley, California, United States) and a krypton/argon laser (643-RYB-A01; Melles Griot, http://lasers.mellesgriot.com). Data processing was performed with Volocity 2.6.1 software (Improvision, http://www.improvision.com).

### Binding of Icm/Dot–secreted L. pneumophila proteins to PIs and other lipids in vitro.

Direct binding of L. pneumophila Icm/Dot–secreted putative effector proteins to PIs and other lipids was tested in a protein–lipid overlay assay [40]. The lipid compounds bound to nitrocellulose membranes were incubated with GST-effector fusion proteins, which were constructed and purified as described above. Preliminary binding experiments were performed using synthetic di-hexadecanoyl–PIs (Echelon Biosciences) or purified authentic di-acyl– (preferentially 1-stearoyl-2-arachidonoyl) PIs (Sigma; Matreya LLC). Diluted stock solutions (3 μl) in CHCl_3_:MeOH:H_2_O = 1:2:0.8 (synthetic PIs) or MeOH (authentic PIs) were spotted onto nitrocellulose membranes yielding 6–200 pmol per spot. The membranes were blocked with 4% fat-free milk powder in TBST (50 mM Tris, 150 mM NaCl, 0.1% Tween-20 [v/v] [pH 8.0]) for 1 h at room temperature and incubated with the fusion proteins (approximately 120 pmol/ml blocking buffer) overnight at 4 °C. Binding of the GST-effector fusion proteins to lipids was visualized by ECL (Amersham Biosciences) using a monoclonal anti-GST antibody (Sigma) and a secondary goat anti-mouse peroxidase-labeled antibody (Sigma). The final experiments were carried out with commercially available PIP-strips and PIP-arrays (Echelon Biosciences), using GST-tagged PH domains of PLCδ1 (PIP_2_ Grip, Echelon Biosciences) and LL*5*α (MultiPIP Grip, Echelon Biosciences) as control reagents for the presence of PI(4,5)P_2_ or all PIs on the nitrocellulose membranes.

To test whether SidC binds to PIs incorporated into PL vesicles, we used affinity-purified GST fusion proteins (GST-SidC or GST-SidD) and commercially available PL vesicles (1 mM lipid) composed of 65% PC, 29% phophatidylethanolamine (PE), 1% biotinylated PE, and 5% either PI(4)P, PI(3)P, or PI(4,5)P_2_ (PolyPIPosomes, Echelon Biosciences). The PL vesicles (20 μl, 1 nmol PI) were incubated for 20 min at 4 °C with GST-SidC or GST-SidD fusion proteins (40 pmol) in a total of 1 ml of binding buffer (50 mM Tris, 150 mM NaCl, 0.05% Nonidet P40 [pH 7.6]). The liposomes were subsequently centrifuged (10 min, 20,800 *g*) and washed five times with 1 ml of binding buffer. Finally, the pellet was resuspended in 25 μl of loading buffer, boiled, and separated on an 8 % SDS polyacrylamide gel. GST fusion proteins were visualized by Western blot with a monoclonal anti-GST antibody (Sigma).

## Supporting Information

Figure S1 A Role for PI3Ks in Intracellular Replication but Not Phagocytosis of Wild-Type L. pneumophila
(A) Phagocytosis by *Dictyostelium* wild-type Ax3 or Δ*PI3K1/2* (untreated or treated with 20 μM LY) of GFP-labeled L. pneumophila wild-type (black bars) or a Δ*icmT* mutant strain (grey bars) was determined by flow cytometry. The data shown are means and standard deviations of duplicates and are representative of at least three independent experiments.
(B) Release of GFP-labeled wild-type *L. pneumophila* from wild-type *Dictyostelium* (filled squares denote untreated; filled triangles denote LY), Δ*PI3K1/2* (open squares denote untreated; open triangles denote LY) was quantified by flow cytometry. As a control for viability, expression of *gfp* by JR32 in the absence (filled diamonds) or in the presence (open diamonds) of LY was determined during the experiment.
(C) Intracellular L. pneumophila quantified by CFU after lysis of *Dictyostelium* with 0.8% saponin. Filled squares denote *Dictyostelium* wild-type Ax3/ JR32; open squares denote Δ*PI3K1/2*/ JR32; filled triangles denote Ax3 + LY/ JR32; filled circles denote Ax3/Δ*icmT;* open circles denote Δ*PI3K1/2*/Δ*icmT;* and open triangles denote Ax3 + LY/Δ*icmT.*
(7.8 MB TIF)

Figure S2 Calnexin or Calreticulin Do Not Affect Intracellular Replication of L. pneumophila within *Dictyostelium*
(A) Release of intracellularly grown L. pneumophila wild-type (denoted by filled symbols) from *Dictyostelium* or killing of Δ*icmT* (denoted by open symbols) by *Dictyostelium* wild-type Ax2 (filled and open squares), or Ax2-derived mutant strains lacking calnexin (filled and open diamonds), calreticulin (filled and open triangles), or calnexin/calreticulin (filled and open circles) is shown. The results were reproduced in four independent experiments.
(B) Release of intracellularly grown L. pneumophila JR32 from wild-type *Dictyostelium* strain Ax3 (filled squares) or Ax3 expressing calnexin-GFP (filled diamonds). The average of two independent experiments, each carried out in triplicate, is shown.
(6.7 MB TIF)

Table S1 Degradation of L. pneumophila Δ*icmT* by *Dictyostelium* Wild-Type Ax3 or Δ*PI3K1/2*
(54 KB DOC)

Table S2 Oligonucleotides Used in This Study (49 KB DOC)

### Accession Numbers

The GenBank (http://www.ncbi.nlm.nih.gov/Genbank) accession numbers for the proteins discussed in this paper are *Dictyostelium* calnexin (AF073837), *Dictyostelium* PI3K1 and PI3K2 (U23476 and U23477, respectively), human FAPP1 (AF286162), L. pneumophila Icm/Dot T4SS conjugation apparatus (Y15044), SidC (AY504673), and SidC paralog SdcA (AY504674).

## Abbreviations

cam: chloramphenicol
CFU: colony-forming unit
CYE: charcoal yeast extract
Dot: defective organelle trafficking
ER: endoplasmic reticulum
FAPP1: phosphatidylinositol(4) phosphate adaptor protein-1
GFP: green fluorescent protein
Icm: intracellular multiplication
IPTG: isopropyl-1-thio-β-d-galactopyranoside
LCV: Legionella-containing vacuole
LY: LY294002
MOI: multiplicity of infection
oligo: oligonucleotide
ORF: open reading frame
PC: phosphatidylcholine
PE: phophatidylethanolamine
PFU: plaque-forming unit
PI: phosphoinositide
PI(4)P: phosphatidylinositol(4) phosphate
PI3K: phosphatidylinositol(3) kinase
PL: phospholipid
RBS: ribosomal binding site
Sid: substrate of intracellular multiplication/defective organelle trafficking transporter
T4SS: type IV secretion system
WM: wortmannin
