# Supplementary material for: Legionella pneumophila Exploits PI(4)P to Anchor Secreted Effector Proteins to the Replicative Vacuole
Source: PLoS Pathog. 2006 May 19;2(5):e46. doi: 10.1371/journal.ppat.0020046 (PMC1463015; doi:10.1371/journal.ppat.0020046)
Supplement: Supplementary file 1 [file ppat.0020046.st001.doc]

**Table S1**. Degradation of *L. pneumophila* Δ*icmT* by *Dictyostelium* wild-type Ax3 or Δ*PI3K1/2*.

|  |  | **Intracellular bacteria ^a^** | | |  |  | |
| --- | --- | --- | --- | --- | --- | --- | --- |
| **Strain** | **Ax3** | |  | **Δ*PI3K1/2*** | | |  |
| **MOI** | **100** | **500** | | **100** | | **500** | |
| 0 d | 44% | 70% | | 10% | | 25% | |
| 1 d | 10% | 32% | | 5% | | 19% | |
| 4 d | 4% | 14% | | 2% | | 10% | |

^a^ “Intracellular bacteria” corresponds to the percentage of *Dictyostelium* amoebae harboring GFP-labeled Δ*icmT*.

The Dictyostelium mutant strain Δ*PI3K1/2* is impaired in phagocytosis and degradation of *L. pneumophila* Δ*icmT*, as determined by the decrease in GFP fluorescence within the *Dictyostelium* scatter gate over time. The experiment was reproduced twice with similar results.
