# Supplementary material for: Legionella pneumophila Exploits PI(4)P to Anchor Secreted Effector Proteins to the Replicative Vacuole
Source: PLoS Pathog. 2006 May 19;2(5):e46. doi: 10.1371/journal.ppat.0020046 (PMC1463015; doi:10.1371/journal.ppat.0020046)
Supplement: Supplementary file 2 [file ppat.0020046.st002.doc]

**Table S2.** Oligonucleotides used in this study.

________________________________________________________________________

Relevant characteristics or sequence *^a^*	Comments

________________________________________________________________________

**Oligonucleotides**

oCR1	AAAAACGCGGATCCATGGTGATAAACATGGTTGACG	5’ *sidC*; *Bam*HI

oCR2	AAAAACGCGTCGACCTATTTCTTTATAATTCCCGTGTAC	3’ *sidC*; *Sal*I

oCR4	AAAAACGCGTCGACTTAAATAGTAAGACTCGAGTTAG	3’ *sidD*; *Sal*I

oCR7	AAAAACGCGTCGACTCATGCTACTATTAAGCATAGAGG	5’ *sdhB*; *Sal*I

oCR8	AAAAGAATGCGGCCGCTTACAATTTGGTAAATTCGATTTCAC	3’ *sdhB*; *Not*I

oCR13	AAAAACGCGGATCCATGCGTTCGATTATTACACAAATC	5’ *sidD*, *Bam*HI

oCR29	AAAAACGCGTCGACTCATGAACATGGTTGACAAAATAAAATTC	5’ *sdcA*; *Sal*I

oCR30	AAAAGAATGCGGCCGCCTATATTGTATTCCTAACAGTTTCTC	3’ *sdcA*; *Not*I

oCR-P1	TATGGCCATGGATCGGAGTAGGGATCGCCTACCTCCTTTTGA-

GACAGAGACGCGTATCCTCGGTGGTGGTGGTGGTG	‘*Nde*I, *Bam*HI’

oCR-P2	GATCCACCACCACCACCACCGAGGATACGCGTCTCTGTCTCA-

AAAGGAGGTAGGCGATCCCTACTCCGATCCATGGCCA	‘*Bam*HI, *Nde*I’

________________________________________________________________________

*^a^* Restriction sites are underlined.
